# Strenuous Exercise Induced Syncope Due to Coronary Artery Anomaly

**Published:** 2014-09-01

**Authors:** Veysel Yavuz, Nurulah Cetin, Esref Tuncer, Onur Dalgic, Ugur Taskin, Ali Riza Bilge, Hakan Tikiz

**Affiliations:** 1 Cardiology Department, Akhisar State Hospital, Manisa, Turkey; 2 Cardiology Department, Ercis State Hospital, Van, Turkey; 3 Cardiology Department, Central Hospital, Izmir, Turkey; 4 Cardiology Department, Turkan Ozilhan State Hospital, Izmir, Turkey; 5 Cardiology Department, Celal Bayar University, Manisa, Turkey

**Keywords:** Syncope, Coronary Anomaly, Coronary Angiography

## Abstract

Coronary artery anomalies are among the neglected topics in cardiology. Anomalous origin of the left main coronary artery from the right sinus of valsalva is a rare coronary anomaly observed in 0.15% of patients. During exercise, the distended aorta and pulmonary artery with increased blood flow may squeeze the Left Main Coronary Artery (LMCA) between them. Even though arrhythmias are common causes of syncope, one should also think about aberrant coronary artery in the patients with syncope of unexplained origin. Patients experiencing exercise induced syncope accompanied by symptoms of coronary ischemia (typically: chest pain, ischemic findings on ECG, and raised cardiac markers) should be referred to diagnostic coronary angiography.

## 1. Introduction

Coronary artery anomalies are among the neglected topics in cardiology. Anomalous origin of the left main coronary artery from the right sinus of Valsalva is a rare coronary anomaly observed in 0.15% of patients (1). Depending on the anatomic relationship between the anomalous vessel and the aorta and the pulmonary trunk, the anomaly may be classified into 4 groups according to the course of the artery; i.e., posterior, interarterial, anterior, and septal courses. Interarterial or preaortic course can be associated with myocardial ischemia and sudden cardiac death (2).

## 2. Case Report

### 2.1. Case 1

**Figure 1.**
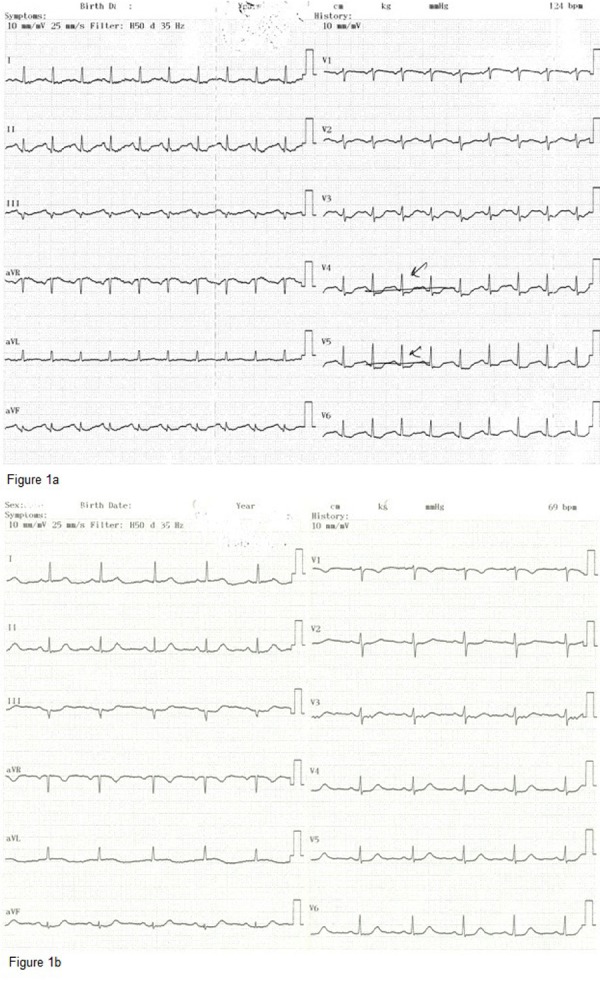
Figure 1a. The Initial ECG of Case 1; The Results Revealed Sinus Tachycardia at 124 bpm and ST Depression in V3 - V6 and DI-AVL Derivations Figure 1b. Control ECG of Case 1; Heart Rate Was Reduced to 69 bpm and ST Depressions Were Completely Resolved on the ECG

**Figure 2.**
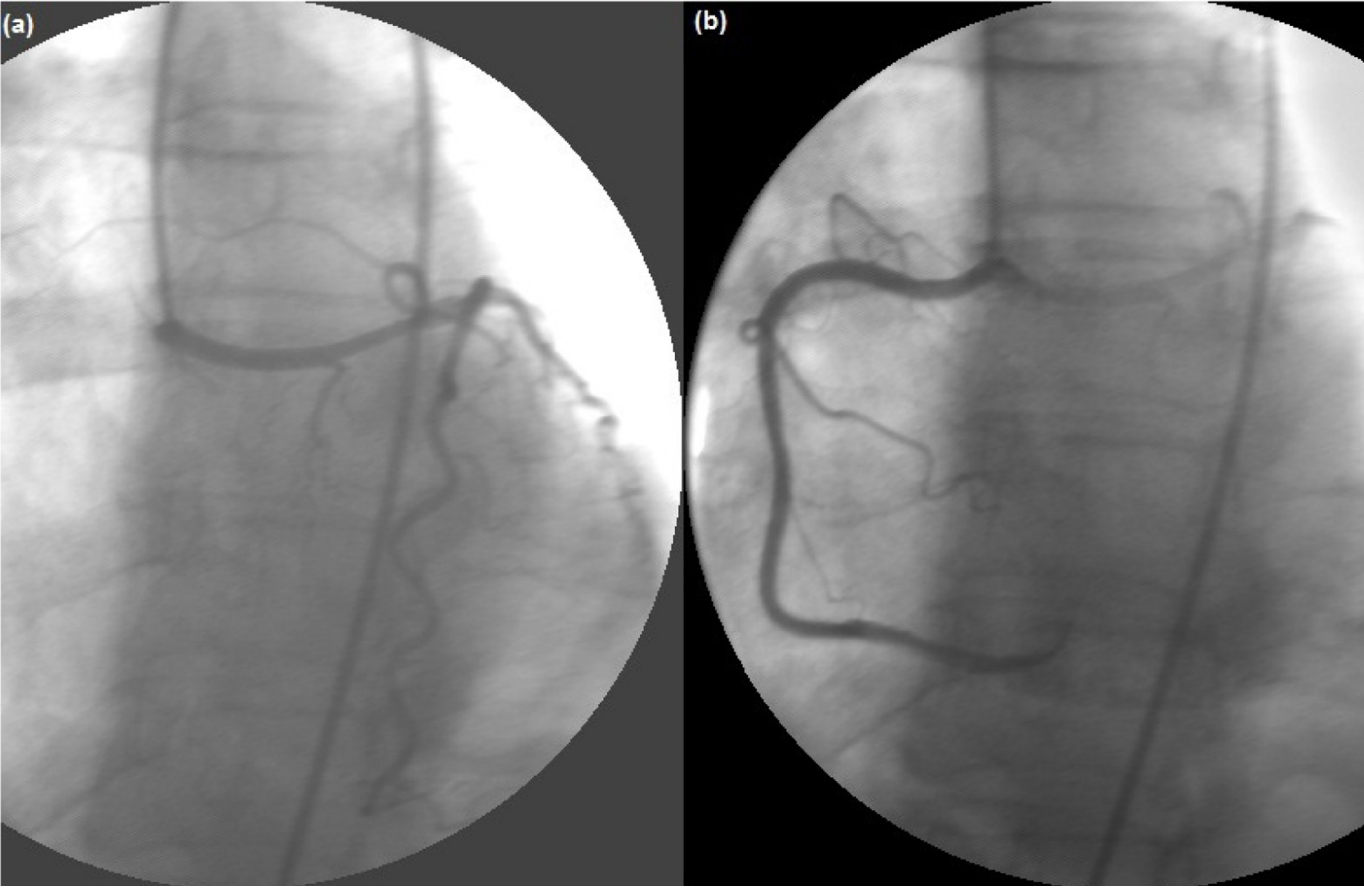
(a) Aberrant Left Main Coronary Artery (LMCA) Arising from the Right Sinus of Valsalva (b) Her Right Coronary Artery Was Arising from the Right Sinus of Valsalva Next to the Emergence of LAD through a Separate Ostium

A 64 year old woman experienced syncope (lasting for one minute), accompanied by chest pain 3 - 4 times after streneous exercise last year. She was referred to our clinic for persistent chest pain after her last syncope. She had a blood pressure of 160/80 mmHg with a rapid, regular cardiac rhythm and a grade 1/6 systolic murmur was heard at the apex. Medical history only revealed hypertension treated by a combination of irbesartan + hydroclorothiazide and lercanadipin. The ECG revealed sinus tachycardia at 124 bpm and ST depression in V3 - V6 and DI-AVL derivations (Figure 1a). One hour later, her heart rate was reduced to 69 bpm and ST depressions were completely resolved on the ECG (Figure 1b). She had mild left ventricular concentric hypertrophy with normal systolic function and minimal mitral regurgitation on echocardiography. Besides, she had increased cardiac enzymes with troponin was 3.88 ng/mL (Normal range < 0.04 ng/mL) and CK-MB was 35.4 U/L (Normal range 0 - 25 U/L). Thus, she was referred for diagnostic coronary angiography for acute coronary syndrome. She had an aberrant Left Main Coronary Artery (LMCA) arising from the right sinus of valsalva (Figure 2a). Her right coronary artery was arising from the right sinus of valsalva next to the emergence of Left Anterior Descending (LAD) artery through a separate ostium (Figure 2b).

### 2.2. Case 2

**Figure 3.**
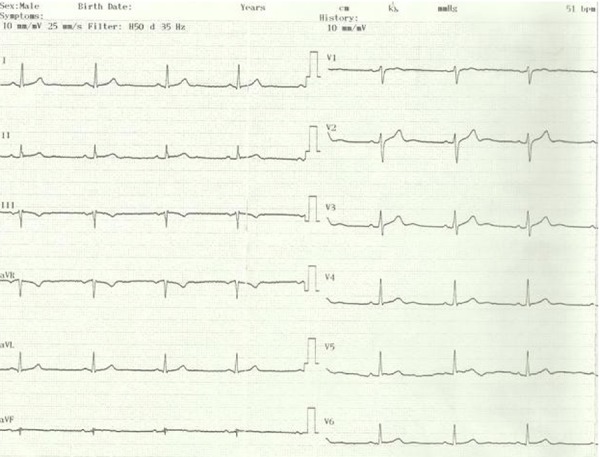
ECG of Case 2, The Patient Had Sinus Bradycardia at 55 bpm and Negative T Waves in DIII and aVF Derivations on ECG

**Figure 4,:**
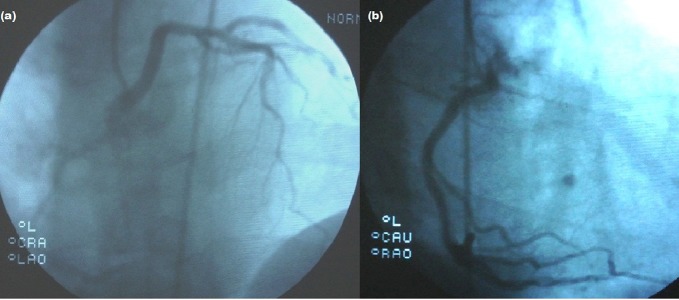
(a) Coronary Angiography Demonstrated an Anomalous Left Main Coronary Artery (LMCA) Arising from the Right Sinus of Valsalva (b) Right Coronary Artery (RCA) Arose from Appropriate Sinus but Different Origin

A 50 year old man experienced syncope after rigorous exercise lasting for about 1 minute and resolving spontaneously for 5 years. His last syncope was accompanied by persistent chest pain. He was referred to our center with acute coronary syndrome. On admission, he had mild but persistent chest pain. He was conscious and had bardycardia (50 bpm). Additionally, his blood pressure was 165/90 mmHg. His medical history revealed hypertension treated with perindopril and amlodipin. He had sinus bradycardia at 55 bpm and negative T waves in DIII and aVF derivations on ECG (Figure 3). Also, he had increased troponin value (1.23 ng/mL), while his CKMB was within the normal range. He had mild left ventricular concentric hypertrophy with normal systolic function on echocardiography. He was referred to coronary angiography due to persistent chest pain. Accordingly, he had an aberrant LMCA arising from the right sinus of valsalva (Figure 4a). The right coronary artery was emerging from a separate ostium on the right sinus of valsalva (Figure 4b).

**Figure 5.**
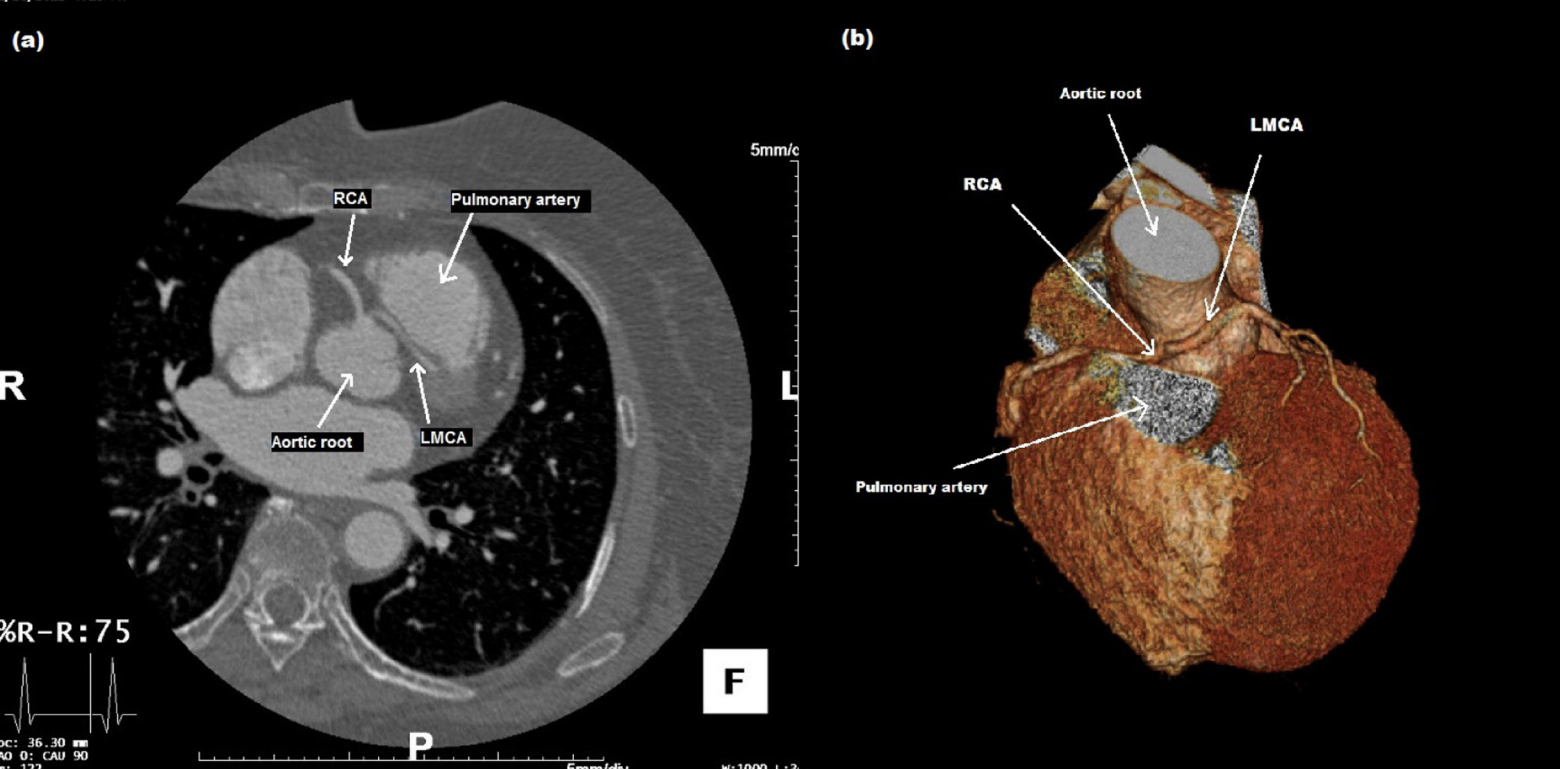
Multidetector Computed Tomography (MDCT); LMCA Coursed between the Aortic Root and Main Pulmonary Artery and Gave off the Left Anterior Descending and Left Circumflex Arteries in Their Normal Position.** (a)** Female Patient (Case 1), **(b)** Male Patient (Case 2)

Both patients underwent cardiac multislice computed tomography to reveal the exact course and location of the aberrant coronary artery. The left main coronary artery was taking a path between the ascending aorta and pulmonary trunk before the emergence of LAD and Cx arteries (Figures 5a - 5b). After all, both patients were referred to cardiac surgery department.

## 3. Discussion

During exercise, distended aorta and pulmonary artery with increased blood flow may squeeze the LMCA between them. Another mechanism of ischemia may be the sharp angle of LMCA after its emergence from the right sinus of valsalva restricting the coronary blood flow (3). Hypertension increases the aortic stiffness and diameter (4). Increase of the sympathetic nervous system activity during exercise further increases aortic stiffness leading to compression of LMCA between the aorta and the main pulmonary artery (5). Syncope of unexplained origin may be a symptom of the presence of an aberrant coronary artery and can be lethal during or after strenuous physical activity (6).

Even though arrhythmias are common causes of syncope, one should also think about the aberrant coronary artery in the patients with syncope of unexplained origin. Patients experiencing exercise induced syncope accompanied by symptoms of coronary ischemia (typically: chest pain, ischemic findings on ECG, and raised cardiac markers) should be referred to diagnostic coronary angiography.
